# 术前D二聚体水平可以预测非小细胞肺癌患者术后1年内预后不良

**DOI:** 10.3779/j.issn.1009-3419.2011.06.10

**Published:** 2011-06-20

**Authors:** 哲 王, 军科 付, 冬梅 刁, 诚学 党

**Affiliations:** 1 710061 西安，西安交通大学第一附属医院胸外科 Department of Toracic Surgery, the First Afliated Hospital of Xi'an Jiaotong University, Xi'an 710061, China; 2 710061 西安，西安交通大学第一附属医院肿瘤外科 Department of Oncologic Surgery, the First Afliated Hospital of Xi'an Jiaotong University, Xi'an 710061, China

**Keywords:** 肺肿瘤, D二聚体, 预后, Lung neoplasms, D-dimer, Prognosis

## Abstract

**背景与目的:**

部分非小细胞肺癌（non-small cell lung cancer, NSCLC）患者术后短期即出现转移。D二聚体（D-dimer, DD）是肺癌（主要是非手术）患者的预后因素。本研究旨在探讨其能否预测NSCLC患者术后短期预后不良。

**方法:**

检测56例NSCLC患者的术前DD水平，术后随访1年，终点是出现预后不良事件（转移、局部复发或肺癌相关性死亡）。统计分析组间不良预后对比情况。

**结果:**

以术前DD水平的中位数（四分位间距）1.05（0.55）mg/L为分界值，高DD组患者中出现预后不良事件11例，而低DD组中出现预后不良事件3例，两组相比具有统计学差异（*P*=0.03, OR=4.89, 95%CI: 1.2-20.1）。高DD组与低DD组的疾病进展情况亦有统计学差异（*P*=0.024）。术前DD对于早期NSCLC（Ⅰ期、Ⅱ期），特别是早期腺癌术后短期预后不良的预测最为准确。

**结论:**

术前DD水平可以预测NSCLC患者术后1年内预后不良，有助于筛选适合手术的患者。

## Abstract (en)

**Background and objective:**

Some operable non-small cell lung cancer (NSCLC) patients have poor prognosis shortly after the surgery. D-dimer (DD) is an independent prognosis factor of lung cancer, especially for inoperable patients. The aim of the study is to investigate whether the pre-operative plasma DD level could predict the poor prognosis shortly after the surgery in operable NSCLC patients.

**Methods:**

The pre-operative plasma DD level of 56 newly diagnosed NSCLC patients without metastasis was examined. All the patients had been followed for one year post-operatively and the end-point was the occurrence of the poor prognosis incident including any sign of the metastasis, local recurrence or death related with the lung cancer. Difference of prognosis according to pre-operative plasma DD level was compared by Chi-square test. Diseases progress was analyzed by *Kaplan-Meier* method.

**Results:**

Among 56 NSCLC patients, 91% had received the curative resections (44 lobectomy and 7 pneumonectomy). There were still 2 cases of the wedge resection and 3 cases of the exploration. The median of the pre-operative plasma DD level was 1.05 (0.55) mg/L. The patients were allocated into two subgroups by the median of the DD levels. There were 11 patients with poor prognosis within one year after the resection in the high DD subgroup, while 3 patients in the low DD subgroup (*P*=0.03, OR=4.89, 95%CI: 1.2-20.1). The diseases progress curves were significantly different between the high and low subgroups (*P*=0.024). Based on plasma DD level, the poor prognosis incident within one year after the surgery was best predicted in the early stage (Ⅰ, Ⅱ) of the NSCLC, especially in adenocarcinoma patients.

**Conclusion:**

The pre-operative plasma DD levels may predict the poor prognosis within one year after the surgery in NSCLC. The measurement of the fibrinolysis marker may help to exclude the unfit patients for the surgery.

恶性肿瘤患者中的凝血纤溶系统常常被异常激活^[1-4]^。不仅静脉血栓的风险增加，肿瘤侵袭转移的能力也大大加强^[5]^。D二聚体（D-dimer, DD）是纤维蛋白降解的最小产物，快速方便的检测以及高度的敏感性，使其成为目前应用最广泛的排除血栓性疾病的纤溶指标^[6, 7]^。临床研究发现DD异常增高的肺癌患者预后不佳^[8]^，可以作为远期预后的指标^[9-11]^，但相关研究主要针对非手术晚期肺癌患者。目前临床上存在一部分非小细胞肺癌（non-small cell lung cancer, NSCLC）患者，各项检查符合手术条件，但术后短期即出现转移甚至死亡。本研究旨在探讨术前DD水平能否预测NSCLC患者术后短期预后不良。

## 1 材料与方法

### 1.1 研究设计

本研究将检测NSCLC患者的术前DD水平，并且术后随访1年。研究终点为出现预后不良事件（poor prognosis incident, PPI），包括局部复发或转移，或出现肿瘤相关性死亡。结合随访资料、DD水平、分期及组织学类型，评价DD的检验价值。

### 1.2 患者资料

2008年1月-2008年12月间西安交通大学第一附属医院胸外科收治的病理学证实的NSCLC患者。每个患者都常规接受了一系列术前检查，包括胸、上腹部CT，全身骨扫描，部分高危病人行头颅CT或MRI，均无明显转移征象。同时心肺功能检查能耐受手术。口服阿司匹林的患者须停药1周以上。患者有血栓史或正在接受抗凝药物者被排除。患者需签署知情同意书。

### 1.3 DD水平检测

清晨空腹采取静脉血，放入含有3.28%的枸橼酸钠的抗凝管。采用增强免疫投射比浊法测定血浆DD水平，DD单克隆抗体购自日本第一试剂公司（Daiichi Seiyaku Co. Ltd. Japan）。

### 1.4 随访

术后根据NCCN NSCLC临床实践指南（2009中国版），部分患者接受了相应术后辅助化疗。术后1年内，每三个月患者门诊随访，包括问诊、全身查体、胸部、上腹部CT，有可疑症状行头颅MRI和骨扫描检查。有气短、咳嗽、疼痛等症状随时回访。部分外地病人在当地检查，均电话随访。

### 1.5 统计处理

采用SPSS 13.0统计软件进行数据分析，术前DD为非正态分布，使用中位数（四分位间距）来描述。采用卡方检验分析组间的不良预后情况，*Kaplan-Meier*法分析疾病进展情况。*P* < 0.05为有统计学差异。

## 2 结果

### 2.1 患者基本资料

入组共56例NSCLC手术患者，中位年龄59岁，年龄范围31岁-81岁，其中男性患者75%。术后病理分期：Ⅰ期13例（23%），Ⅱ期22例（39%），Ⅲ期18例（32%），Ⅳ期3例（5%）；组织学类型：腺癌27例（48%）、鳞癌23例（41%）、腺鳞癌3例、未分化癌1例、肉瘤样癌1例、类癌1例；手术方式：肺叶切除44例（79%）、全肺切除7例（12%）、楔形切除2例、开胸探查3例。所有患者均行纵膈淋巴结清扫或采样。

### 2.2 术后随访资料

在术后1年的随访期内，出现疾病进展或肿瘤相关性死亡的患者共14人，术中发现胸膜广泛转移3例、皮下转移1例（术后3周，肉瘤样癌）、脑转移2例（分别在术后3个月、4个月）、骨转移2例（分别在术后3个月、9个月）、双肺转移1例（术后6个月）、恶性胸水3例（分别在术后1个月、3个月、4个月）、化疗期间肺栓塞死亡1例（术后3个月）、局部复发1例（术后12个月）。除死亡患者，1年后无失访患者。

### 2.3 DD数据分析

56例患者的术前DD值为非正态分布，中位数（四分位间距）为1.05（0.55）mg/L。以中位数1.05 mg/L为高低组分界值，高DD组共29例，其中术后1年内出现预后不良事件11例（38%），低DD组27例中术后1年内出现预后不良事件3例（11%），两组相比具有统计学差异（*P*=0.03, OR=4.89, 95%CI: 1.2-20.1）。高DD组与低DD组的疾病进展情况亦有统计学差异（*P*=0.024）（图 1）。

**1:**
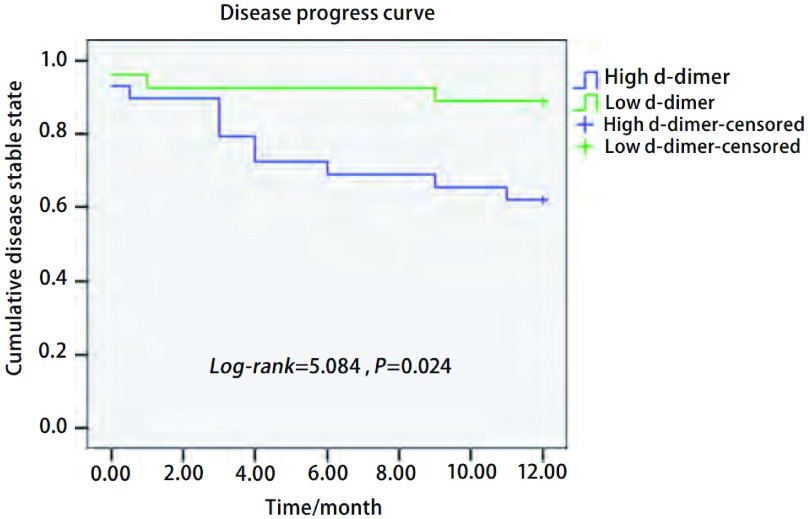
术前高D二聚体组与低D二聚体组的疾病进展曲线（根据出现术后不良预后事件的时间） The disease progress curves of the high and low d-dimer groups (According to the time of the occurrence of the poor prognosis incident)

### 2.4 DD检验效率

术前DD水平（中位数）预测NSCLC患者术后1年内预后不良事件的敏感性为79%，特异性为57%，阳性预测值为38%，阴性预测值为89%。如果参考组织学类型及TNM分期，对Ⅰ期、Ⅱ期病变的检验效率更高，敏感性和阴性预测值均为100%，其中对于腺癌的特异性较鳞癌更高；对Ⅲ期病变，检验效率明显下降。其中对于鳞癌在敏感性和阴性预测值方面好于腺癌，特异性和阳性预测值均不理想（表 1）。

**1:** 术前D二聚体（中位数）预测NSCLC患者术后1年内预后不良事件的检验效率 The efficiency of the pre-operative d-dimer (median) predicting the poor prognosis incident within 1 year after the operation in NSCLC

Efficiency	NSCLC	Squamous cell carcinoma			Adenocarcinoma	
		(Ⅰ, Ⅱ)	Ⅲ		(Ⅰ, Ⅱ)	Ⅲ
Sensitivity	79% (11/14)	1 DO% (3/3)	100% (1/1)		100% (1/1)	50% (2/4)
Specificity	57% (24/42)	43% (7/16)	33% (1/3)		86% (12/14)	60% (3/5)
Positive predictive value	38% (11/29)	30% (3/10)	33% (1/3)		33% (1/3)	50% (2/4)
Negative predictive value	89% (24/27)	100%	100%		100%	60% (3/5)
NSCLC: non-small cell lung cancer. ^*^Being a predictor, the d-dimer (median) does well in the Sensitivity and the Negative predictive value. The predictive efficiency is highest among the adenocarcinoma patients in the (Ⅰ, Ⅱ) stage, and lowest among the adenocarcinoma patients in the Ⅲ stage. ^*^The undifferentiated carcinoma, adenosquamous carcinoma and the carcinoma sarcomatodes were not analyzed for too small patient numbers.						

## 3 讨论

19世纪Trousseau从胃癌患者出现游走性血栓静脉炎的现象中发现了恶性肿瘤患者中广泛存在凝血纤溶功能的障碍^[12]^，而后恶性肿瘤与凝血纤溶系统两者的相互关系逐渐被广泛重视^[13]^。肺癌引起高凝状态的机制可能与其过度表达组织因子（tissue factor, TF）有一定关系^[14-16]^，许多凝血纤溶指标如PT、APTT、纤维蛋白原，Ⅱ、Ⅴ、Ⅷ因子，纤维蛋白原降解产物（fibrin degradation products, FDP），以及血凝酶-抗血凝酶Ⅲ复合物（thrombin-antithrombin Ⅲ complexes, TAT）都可以出现异常^[17, 18]^。同时激活的凝血系统也可以促进肿瘤的播散和转移^[13, 19]^。所以伴有高凝状态的恶性肿瘤常常表现出巨大的瘤负荷，肿瘤进展活跃，化疗反应率低，以及最终预后差的临床特点^[20]^。而抗凝治疗在一部分肿瘤中被证实具有抑制肿瘤生长转移的效果^[21, 22]^。

DD是纤维蛋白降解的最小产物，可以敏感地反映出纤溶系统的激活情况。目前临床上广泛用于血栓性疾病的排除诊断。DD是众多凝血纤溶指标中，与肺癌患者的疾病进展，生存时间，化疗效果相关性最好的指标^[23, 24]^。但令人遗憾的是，在可手术的早期肺癌患者中，DD的临床意义研究不多^[25]^，而同时结合患者预后分析的研究更少。

如何能判断患者接受肺癌手术后的效果是一个复杂的问题，影响因素很多。年龄、体力状况、体重变化、术后病理分期是4个经典的判断预后的指标^[9]^。但临床上存在根据术后pTNM分期所预期的效果与实际术后疗效大相径庭的情况, 特别是部分患者术后早期即出现的不良预后，似乎完全无法用病理分期解释。医生需要更佳的预测指标来帮助判断术后效果。一项研究^[26]^发现，腺癌、CEA的水平以及PET检查中SUV值均是独立于pTNM分期预测早期肺癌术后复发的影响因素。本研究结果中术前DD的异常增高提示患者术后效果不佳，术后1年内出现预后不良事件（疾病进展或肿瘤相关性死亡）的风险明显增加（*P*=0.03, OR=4.89, 95%CI: 1.2-20.1)。

TNM分期对于远期预后的影响是毋庸置疑的，但术后短期内出现的预后不良事件在发生机制上似乎与远期预后不良有些差异。它多为术前业已存在常规检查却无法发现的微转移，或是由于肺癌高凝状态引起的血栓事件，而病理分期对于上述机制几乎无法评估。如果术前常规检查判断适合手术，则提示根据TNM分期与预后的关系，术后短期内不该出现预后不良事件，起码概率很低。但事实上临床上的确存在这样的小概率事件，并且还往往是医疗纠纷的根源。对此，经典的TNM分期体系几乎无能为力。而根据我们的结果，DD可以判断手术后短期预后不佳。它独立于TNM分期体系，是其有益的补充。本组肺癌Ⅱ期患者中18%（4/22）和Ⅲ期患者中38%（7/18）术后1年内出现预后不良事件，无法从手术中获益。如果使用术前DD来判断，可以预测全体NSCLC患者中79%的不良预后事件。结合特异性、阳性预测值和阴性预测值，对于早期（Ⅰ期、Ⅱ期）腺癌，DD的预判效果最佳，而中晚期（Ⅲ期）腺癌预判效果最差，鳞癌介于两者之间。目前最大一项评估DD在肺癌中意义的研究^[9]^结果提示在早期肺癌和腺癌患者中DD预测效率更高。而本研究中DD在中晚期腺癌中的预测效率却很低，两研究结果有明显差异。分析原因，首先本研究病例数太少，结果需要更大样本量证实，其次东方人和高加索人在肺腺癌基因突变的差异也应该被考虑。

术中探查发现广泛胸膜转移的病例（Ⅳ期）是术后短期预后不良事件的特例，通过DD来预测，虽然敏感性高，阳性预测值却只有7%，所以没有太多临床意义，只是阴性预测值100%，仅有一定排除意义。本组的3例胸膜转移均为腺癌，也再次提示DD对于晚期腺癌的判断效率较低。

本研究的不足主要是病例数偏少，需要大规模多中心的研究和更长时间的随访来更加充分地评价DD在肺癌手术患者中的价值。另外在分类评估时，本研究主要考虑组织类型和TNM分期，今后的研究应参考基因突变分型等，其结果将更加有意义。

总之，根据本研究的结果，术前DD水平可以预测NSCLC患者术后短期的预后不良。其预测结果在早期NSCLC，特别是早期腺癌患者中最为可靠。
